# Galectins can serve as biomarkers in COVID-19: A comprehensive systematic review and meta-analysis

**DOI:** 10.3389/fimmu.2023.1127247

**Published:** 2023-02-23

**Authors:** Amir Hossein Behnoush, Amirmohammad Khalaji, Seyedeh Yasaman Alemohammad, Amirali Kalantari, Alessandro Cannavo, Charles J. Dimitroff

**Affiliations:** ^1^ School of Medicine, Tehran University of Medical Sciences, Tehran, Iran; ^2^ Non–Communicable Diseases Research Center, Endocrinology and Metabolism Population Sciences Institute, Tehran University of Medical Sciences, Tehran, Iran; ^3^ Robert Stempel College of Public Health & Social Work, Florida International University, Miami, FL, United States; ^4^ Department of Translational Medical Sciences, Federico II University of Naples, Naples, Italy; ^5^ Department of Translational Medicine, Translational Glycobiology Institute at Florida International University, Herbert Wertheim College of Medicine, Florida International University, Miami, FL, United States

**Keywords:** COVID-19, galectin, biomarker, systematic review, meta-analysis

## Abstract

**Background:**

Galectins are an eleven-member class of lectins in humans that function as immune response mediators and aberrancies in their expression are commonly associated with immunological diseases. Several studies have focused on galectins as they may represent an important biomarker and a therapeutic target in the fight against COVID-19. This systematic review and meta-analysis examined the usefulness of clinical assessment of circulating galectin levels in patients with COVID-19.

**Methods:**

International databases including PubMed, Scopus, Web of Science, and Embase were systematically used as data sources for our analyses. The random-effect model was implemented to calculate the standardized mean difference (SMD) and a 95% confidence interval (CI).

**Results:**

A total of 18 studies, comprising 2,765 individuals, were identified and used in our analyses. We found that Gal-3 is the most widely investigated galectin in COVID-19. Three studies reported significantly higher Gal-1 levels in COVID-19 patients. Meta-analysis revealed that patients with COVID-19 had statistically higher levels of Gal-3 compared with healthy controls (SMD 0.53, 95% CI 0.10 to 0.96, P=0.02). However, there was no significant difference between severe and non-severe cases (SMD 0.45, 95% CI -0.17 to 1.07, P=0.15). While one study supports lower levels of Gal-8 in COVID-19, Gal-9 was measured to be higher in patients and more severe cases.

**Conclusion:**

Our study supports Gal-3 as a valuable non-invasive biomarker for the diagnosis and/or prognosis of COVID-19. Moreover, based on the evidence provided here, more studies are needed to confirm a similar diagnostic and prognostic role for Gal-1, -8, and -9.

## 1 Introduction

In December 2019, the coronavirus (CoV) disease of 2019 (COVID-19) first appeared in Wuhan, Hubei Province (1). There are major diagnostic and assessment challenges associated with the COVID-19 pandemic brought on by severe acute respiratory syndrome (SARS) CoV-2 (SARS-CoV2) as the pathogen responsible for the disease. These include figuring out the importance of signs and symptoms in predicting potential infections, determining whether current biochemical and laboratory tests can detect infections and identify patients needing immediate medical attention, and seeing if new diagnostic tests can enable precise rapid and point-of-care testing (2).

In this regard, biomarkers play a crucial role in the clinical decision since they can anticipate the COVID-19 severity and track its prognosis (3). For this reason, in the last few years, several biomarkers have been investigated and identified. For instance, studies have demonstrated that in patients with a higher severity level of COVID-19 pneumonia, increased serum levels of C-reactive protein, interleukin-10, and interleukin-6/Interferon-γ ratio were frequently found (4–6). In addition, others reported that blood levels of D-dimer, lactate dehydrogenase, and white blood cell count played a role in predicting adverse outcomes in the severe COVID-19 (7).

Galectins are a group of lectins with a phylogenetically conserved structure that share approximately 130 amino acids that make up the consensus amino acid sequence, and the carbohydrate recognition domain is in charge of binding β-galactoside (8, 9). Galectin family members have been identified in circulating cells, main and secondary lymphoid tissues, and other organs (10). To date, fifteen mammalian galectins have been discovered and only eleven are found in humans (11). Among these, galectin (Gal)-1, -3, -8, and -9 have been well-described as key immune response mediators. While Gal-1 generally exhibits suppressive activities on the immune system, Gal-3 and -9 have been shown to suppress or amplify inflammatory responses (12). Likewise, Gal-8 has also been suggested to have disparate roles in immunity, functioning as an enhancer of the expression and secretion of many pro-inflammatory cytokines or as a cytokine-binding partner to sequester and hinder cytokine activity (12). For this reason, galectins have been linked to a variety of inflammatory-driven disorders (e.g., cancer, cardiovascular, and metabolic diseases) (11–16). Moreover, since these molecules are also involved in pro-fibrotic processes (13), their role is particularly relevant because they are involved in the pathogenesis of several disorders affecting several organs including the heart and lungs (16–18).

Based on this evidence, recent studies suggest the clinical utility of evaluating Gal-1, -3, -8, and -9 circulating levels as novel potential diagnostic and prognostic biomarkers of COVID-19 either alone or in conjunction with other known biomarkers (19–22). Hence, here we conducted a systematic review and a meta-analysis to combine the findings from previous studies assessing Gal-1, -3, -8, and -9 as putative, non-invasive biomarkers of COVID-19 severity and COVID-19-related complications *(e.g., cardiovascular)*. The conclusions obtained by this study can pave the way for future research on these biomarkers and further enlighten their role in clinical settings.

## 2 Methods

### 2.1 Search strategy

International online databases including PubMed, SCOPUS, Web of Science, and SCOPUS were searched from inception to October 2022 without any filters or limitations. Search queries consisted of keywords related to COVID-19 and galectins. Details of the search strategy are depicted in **Supplementary Table 1**.

### 2.2 Inclusion and exclusion criteria

Inclusion criteria were clinical studies that (1): compared galectin levels between COVID-19 patients and healthy controls (2), compared galectin levels between different severities of COVID-19 (3), compared galectin levels between patients with comorbidity and without comorbidity related to COVID-19, or (4) used galectin levels to discriminate COVID-19 patients from healthy controls or differentiate different severities of COVID-19. We excluded animal studies, case reports, reviews, and conference abstracts. Finally, non-English abstracts were also excluded.

### 2.3 Screening, data extraction, and quality assessment

Required data were extracted by two reviewers (AHB and SYA) in a pre-designed sheet by the third reviewer (AKh). We extracted these for each study (1): study characteristics including publication year, locations(s), and first author’s name (2), population characteristics including total population number, mean age, and female percentage, and (3) main findings.

The Newcastle-Ottawa Scale (NOS) for non-randomized studies was used to assess the risk of bias in included studies (23). NOS evaluates studies according to pre-specified items including selection, comparability, and exposure. Studies with a total NOS ≥ 8 are considered high quality.

### 2.4 Statistical analysis and data synthesis

Data synthesis was performed from the mean and standard deviations (SDs) of galectin levels in COVID-19 cases or different severities of the disease. As there were sufficient studies for Gal-3, we conducted meta-analyses for comparison of this biomarker between COVID-19 cases vs controls, and severe vs. non-severe forms of the disease. Random-effect meta-analysis was performed to calculate the pooled effect size using standardized mean difference (SMD) and 95% confidence interval (CI). To find the source of heterogeneity, meta-regression was conducted with variables including sample size, mean age, and female percentage. The associated bubble plots were also shown. Moreover, publication bias was evaluated using the visual symmetry of funnel plots and statistical tests of Begg’s and Egger’s (24, 25). Finally, to find the possible outliers of analyses, the Galbraith plots were designed, and sensitivity analysis was performed with the removal of each study in analyses.

## 3 Results

### 3.1 Literature search and baseline characteristics of included studies

**Figure 1:**
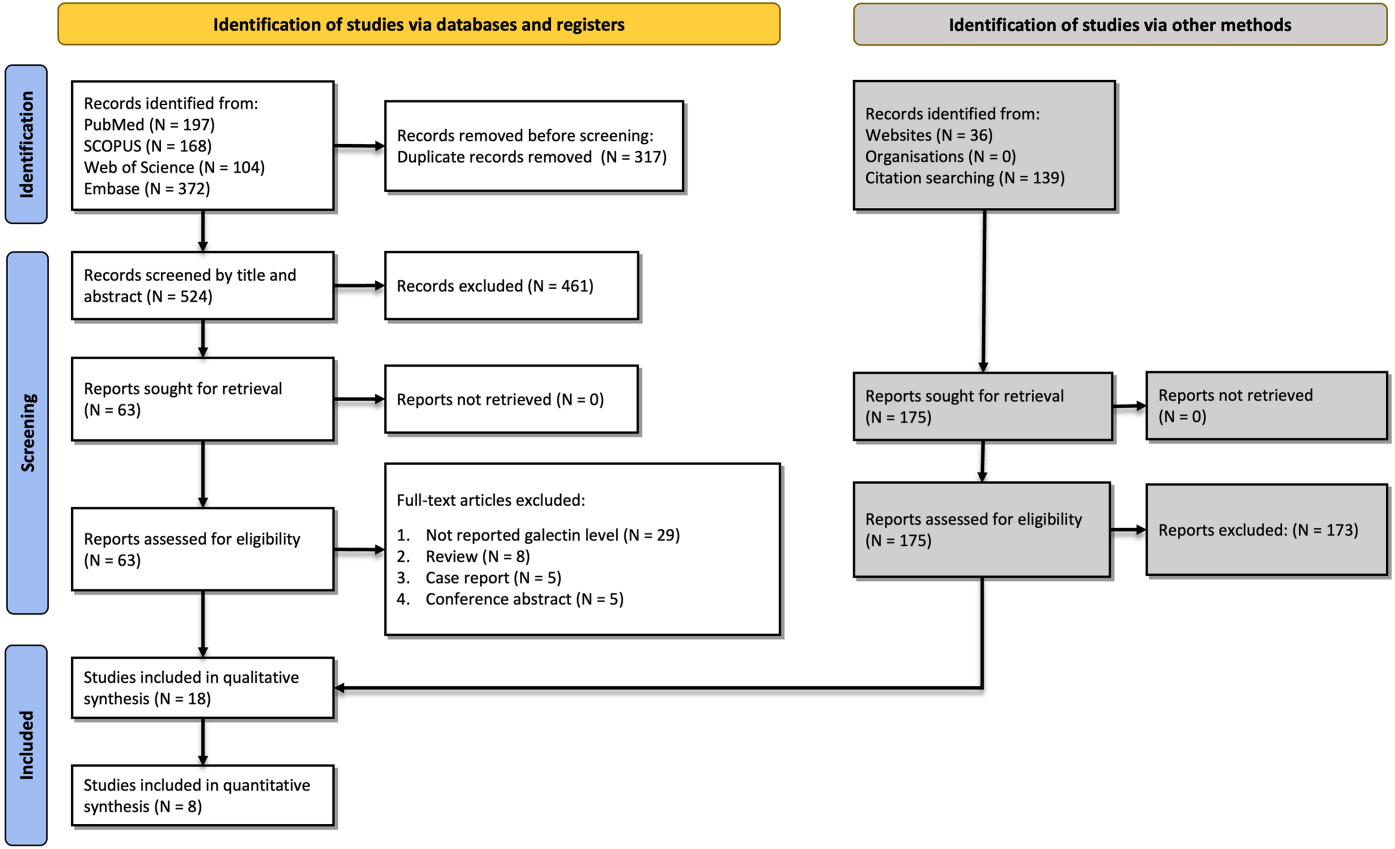
Flow diagram summarizing the selection of eligible studies based on the PRISMA guidelines.

Our search resulted in 841 records: 197 from PubMed, 168 from SCOPUS, 104 from Web of Science, and 372 from Embase. After removing duplicates, 524 records were screened by titles and abstracts. Following this step, 63 studies were evaluated by full texts, resulting in 18 final included studies (7, 19–22, 26–38). The PRISMA (Preferred Reporting Items for Systematic reviews and Meta-Analyses) flow chart summarizing the selection process is available in **Figure 1**.

A total of 2,765 individuals were included between 2020 and 2022. Gal-1 (20, 22, 35), Gal-3 (7, 20–22, 27, 29–34, 36–38), Gal-8 (19), and Gal-9 (19–21, 26, 28) were evaluated in three, 14, one, and five studies, respectively. Twelve studies evaluated serum galectin levels (7, 22, 27, 29–37), while six others evaluated galectins in plasma (19–21, 26, 28, 38). **Table 1** summarizes baseline characteristics and main findings of included studies. All studies had high quality based on NOS (**Supplementary Table 2**).

**Table 1:** Main findings and baseline characteristics of included studies.

Author	Year	Galectin	Location(s)	Specimen	Population	N Total	Female (%)	Age (years)	Main findings
Bai et al.	2021	Gal-9	Japan	Plasma	Mild COVID-19, pneumonia in COVID-19, and bacterial infection	62	32.2	NR	Levels of Gal-9 were significantly higher in patients with pneumonia compared to mild COVID-19. In addition, Gal-9 levels were higher in all COVID-19 patients compared to healthy controls (P<0.05). The prediction of COVID-19 disease with Gal-9 levels was acceptable (AUC>0.88).
Baykan et al.	2021	Gal-3	Turkey	Serum	RT-PCR-positive COVID-19 patients and healthy controls	176	52.8	61.3 ± 13.7	Gal-3 levels in patients with typical pneumonia were significantly higher than in patients with atypical (p<0.01), indeterminate appearance (p<0.01), and patients without pneumonia (p<0.01). Higher Gal-3 levels were significantly associated with higher severity of lung involvement. Prediction of typical pneumonia in COVID-19 patients resulted in an AUC of 0.89 (95% Cl 0.83-94 p:<0.01). A cut-off of 18.9 ng/mL resulted in 87% sensitivity and 73% specificity.
Bozorgmehr et al.	2021	Gal-9	Canada	Plasma	RT-PCR-positive COVID-19 patients and healthy controls	179	NR	NR	Plasma levels of Gal-9 were significantly higher in COVID-19 patients compared to healthy controls. In addition, Gal-9 levels were higher in severe COVID-19 patients compared to mild-to-moderate ones. A cut-off of 2042 pg/mL separated controls from COVID-19 patients with 95% sensitivity and 95% specificity.
Bruni et al.	2022	Gal-3	Switzerland	Serum	RT-PCR-positive COVID-19 patients and RT-PCR-negative patients with signs of COVID-19	319	41.1	57 ± 18.1	COVID-19 patients had insignificant levels of Gal-3 compared to controls (9.3 [7.1-12.3] vs. 8.5 [6.9-10.8]; P = 0.10). In addition, in COVID-19 patients, higher levels of Gal-3 had detected in patients admitted to ICU or patients dead at 30 days (12.1 [10.3-15.9] ng/mL vs. 8.1 [6.5-11.3] ng/mL; P<0.001).
Cannavo et al.	2022	Gal-3	Italy	Serum	RT-PCR-positive hospitalized COVID-19 patients and healthy controls	70	18.0	60.6 ± 13.0	Serum Gal-3 levels were significantly higher in COVID-19 patients compared to healthy controls (P<0.0001). In addition, moderate and severe COVID-19 also showed higher Gal-3 levels compared to healthy controls (P=0.015; P<0.0001, respectively).
Cervantes-Alvarez et al.	2022	Gal-3	Mexico	Serum	RT-PCR-positive COVID-19 patients and age-matched healthy controls	166	NR	53.2 ± 13.2	The Gal-3 level was an independent predictor of invasive mechanical ventilation and/or in-hospital mortality in COVID-19 patients (41.17 [29.71-52.25] vs. 23.76 [15.78-33.97] ng/mL) with an OR of 3.68 [1.47-9.20] in a forward-stepwise logistic regression model (P<0.01).
Chen et al.	2021	Gal-3 and 9	Taiwan, China, and USA	Plasma	RT-PCR-positive COVID-19 patients and healthy controls	86	59.3	44.4 ± 15.1	Plasma Gal-3 and -9 levels were significantly higher in severe COVID-19 patients compared with healthy controls (P<0.001).
De Biasi et al.	2020	Gal-1, -3, and -9	Italy	Plasma	RT-PCR-positive COVID-19 patients and age- and sex-matched healthy controls	64	17.9	NR	Gal-1, -3, and -9 were significantly higher in COVID-19 patients compared with healthy controls.
Ercin et al.	2021	Gal-3	Turkey	Serum	RT-PCR-positive COVID-19 patients and non-symptomatic healthy controls	86	53.5	47.1 ± 17.4	Serum levels of Gal-3 were significantly higher in COVID-19 patients compared with healthy controls (16.7 [13.2-31.5] ng/mL vs. 11.1 [8.5-13.6] ng/mL; P<0.001).
Karsli et al.	2022	Gal-3	Turkey	Serum	RT-PCR-positive COVID-19 patients and non-symptomatic healthy controls	150	60.7	65.2 ± 14.1	Compared to healthy controls (13.57 [10.9-16.4] ng/mL), serum Gal-3 levels were higher in COVID-19 patients. However, Gal-3 levels were significantly lower in severe/critical disease group compared to healthy controls (13.52 [10.69-16.60] ng/mL; P=0.019) and compared to moderate disease group (11.65 [6.09-14.33] ng/mL; P=0.019).
Kazancioglu et al.	2021	Gal-1 and -3	Turkey	Serum	RT-PCR-positive hospitalized COVID-19 patients and healthy controls	140	68.6	NR	Serum levels of Gal-1 were significantly higher in COVID-19 patients compared to healthy controls (P<0.001); however, no significant difference was seen between severe and non-severe groups (P=0.263). Gal-3 levels were also significantly higher in COVID-19 patients compared with healthy controls (P<0.001). Contrary to Gal-1, the severe group had higher Gal-3 levels compared to non-severe group (P=0.011).
Kusnierz-Cabala et al.	2021	Gal-3	Poland	Serum	RT-PCR-positive hospitalized COVID-19 patients	70	61.4	58 ± 13.6	No significant difference was seen in serum Gal-3 levels in COVID-19 patients compared with healthy controls (P>0.05). However, patients with pneumonia had significantly higher levels compared to healthy controls (P=0.009). A positive correlation was detected between Gal-3 levels and length of hospital stay (R=0.28; P=0.023). Gal-3 above 13.51 ng/mL was associated with pneumonia with 52% sensitivity and 86% specificity.
Markovic et al.	2022	Gal-1	Serbia	Serum	RT-PCR-positive hospitalized COVID-19 patients	210	42.6	58.6 ± 12.3	Serum levels of Gal-1 were significantly higher in stage III of COVID-19 compared to stage I and II (P=0.001). A positive correlation between Gal-1 levels and presence of dry cough (r=0.242; P=0.001), headache (r=0.158; P=0.034), and chest radiographic findings (r=0.352; P=0.001). Moreover, a negative correlation was detected between Gal-1 levels and normal breathing sound (r=0.151; P=0.043). Diagnostic accuracy for stage II and stage II detection was acceptable (stage II: cut-off: 3959 pg/mL resulted in sensitivity of 89.5% and specificity of 23.4%; stage III: cut-off: 9359 pg/mL resulted in sensitivity of 72.2% and specificity of 25.9%).
Ozcan et al.	2022	Gal-3	Turkey	Serum	RT-PCR-positive hospitalized COVID-19 patients	175	44	55.5 ± 15.4	Gal-3 levels were significantly higher in severe COVID-19 patients compared to non-severe ones (1.07 ± 0.75 vs. 0.484 ± 0.317; P<0.0001). High Gal-3 level was also associated with higher in-hospital mortality, need for advanced ventilatory support, and need for ICU admission.
Portacci et al.	2021	Gal-3	Italy	Serum	RT-PCR-positive COVID-19 patients admitted to ICU with ARF	140	30	67.3 ± 13.4	Serum Gal-3 level higher than 35.3 ng/mL was associated with higher mortality, ICU admission, and severe ARDS.
Rodriíguez-Tomàs et al.	2021	Gal-3	Spain	Serum	RT-PCR-positive hospitalized COVID-19 patients, RT-PCR-negative patients, and healthy controls	221	38.5		Gal-3 levels were higher in COVID-19 patients compared to healthy controls (P<0.0001). However, it was not a good marker to discriminate RT-PCR-positive and RT-PCR-negative individuals (AUC=0.709).
Tawiah et al.	2022	Gal-3	United States	Plasma	RT-PCR-positive COVID-19 patients admitted to ED	358	41.3	59.8 ± 17.1	Plasma levels of Gal-3 were higher in patients who died 30 days after admission (55.5 [41.2-87.0] ng/mL vs. 39.0 [25.4-58.5]). In addition, Gal-3 predicted the 30-day mortality with an AUC of 0.68 [0.60-0.76]. A log-2 unit increase in Gal03 level was associated with a 92% increased mortality risk.
Yasar et al.	2021	Gal-8 and -9	Turkey	Plasma	RT-PCR-positive COVID-19 patients and healthy controls	93	36.6	58.6 ± 15.3	Gal-9 levels increased with higher severity of COVID-19 in addition to higher levels compared to healthy controls (P<0.0001). However, COVID-19 patients had lower levels of Gal-8 compared to healthy controls (P<0.0001).

Data are presented as mean ± standard deviation, median [interquartile range], or percentage.

Gal, galectin; COVID-19, coronavirus disease of 2019; RT-PCR: Reverse transcriptase polymerase chain reaction; ED, Emergency department; NR, Not reported; AUC, area under the curve; ICU: intensive care unit; ARDS, acute respiratory distress syndrome; ARF, acute respiratory failure.

### 3.2 Gal-1 and COVID-19

#### 3.2.1 Gal-1 levels comparison between COVID-19 patients and controls

The association between Gal-1 levels and COVID-19 was evaluated by three studies (20, 22, 35). In a case-control study by De Biasi et al. (20), Gal-1 levels were compared between 39 COVID-19 patients and 25 age- and sex-matched healthy controls. They found significantly higher levels of Gal-1 in COVID-19 patients compared to healthy controls. In line with this study, Kazancioglu et al. (22) found higher serum levels of Gal-1 in COVID-19 patients compared to healthy controls (P < 0.001).

#### 3.2.2 Gal-1 levels in different severities of COVID-19

The study by Kazancioglu et al. (22) found no significant difference between severe (n = 29) and non-severe (n = 55) COVID-19 patients (median = 9.86 vs. 6.35 ng/mL). In contrast, Markovic et al. (35) found higher levels in stage III of COVID-19 compared to stages I and II (P = 0.001). Detection of stage III COVID-19 was possible with a cut-off of 9359 pg/mL (specificity: 25.9% and sensitivity: 72.2%). In addition, stage II was also associated with higher levels of Gal-1 compared to stage I (P < 0.05) and detection of stage II COVID-19 was possible with a cut-off of 3959 pg/mL (specificity: 23.4% and sensitivity: 89.5%).

#### 3.2.3 Gal-1 levels and COVID-19 complications

Markovic et al. (35) was the only study that investigated Gal-1 levels with COVID-19 complications. The correlation between Gal-1 levels and dry cough (r = 0.242; P = 0.001), headache (r = 0.158; P = 0.034), and chest radiographic findings (r = 0.352; P = 0.001) was positive. However, a negative correlation between normal breathing sound and Gal-1 levels was also seen (r = 0.151; P = 0.043).

### 3.3 Gal-3 and COVID-19

#### 3.3.1 Meta-analysis of Gal-3 levels in COVID-19 vs. controls

**Figure 2:**
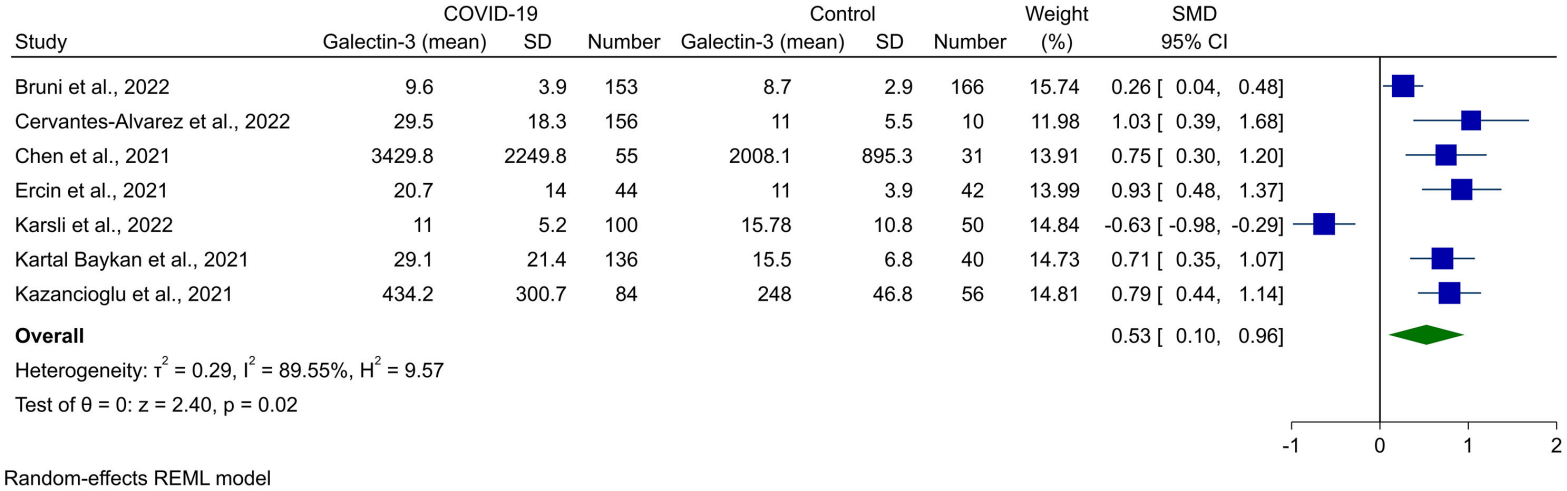
Forest plot for the meta-analysis of Gal-3 levels in COVID-19 patients vs. healthy controls.

**Figure 3:**
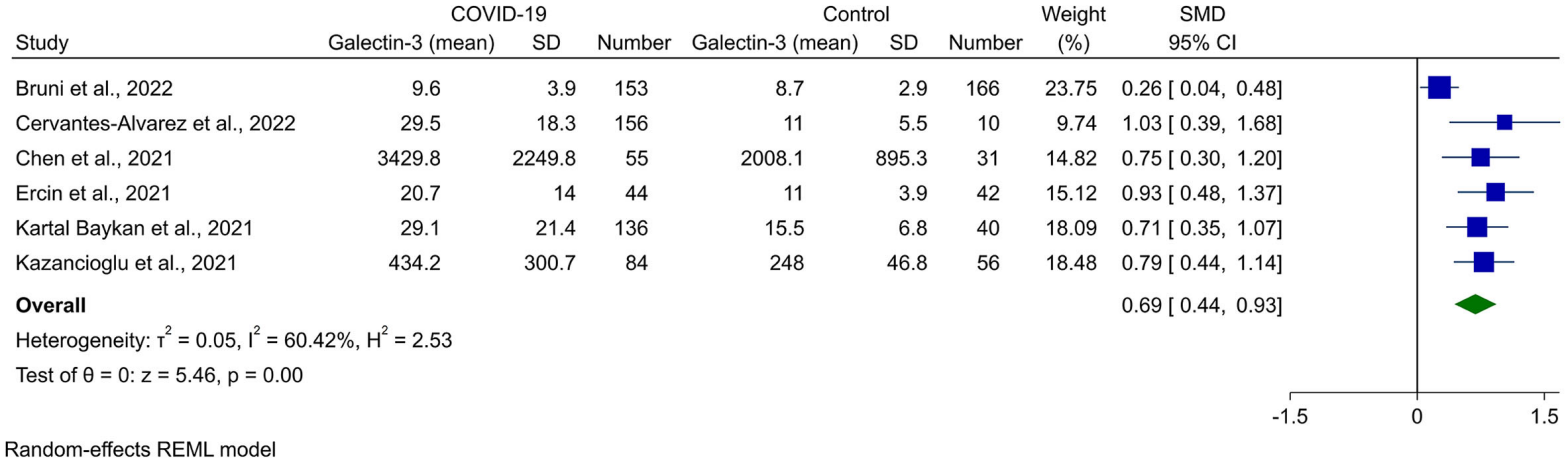
Forest plot for the meta-analysis of Gal-3 levels in COVID-19 patients vs. healthy controls after removing the outlier study (33).

Random-effects meta-analysis of seven studies, comprised of 1,123 cases (728 COVID-19 and 395 controls), showed that Gal-3 levels were significantly higher in COVID-19 cases compared to healthy controls (SMD 0.53, 95% CI 0.10 to 0.96, P = 0.02, **Figure 2**). This was associated with high heterogeneity (*I* ^2^ = 89.55%). The outliers for this analysis were recognized using the Galbraith plot, which is shown in **Supplementary Figure 1**. After removing Karsli et al. (33), no change was observed in the significance of the results (SMD 0.69, 95% CI 0.44 to 0.94, P < 0.01, **Figure 3**), although heterogeneity was reduced to 60.42%. Removing five of the studies made the overall result insignificant (21, 22, 27, 31, 32).

Meta-regression was performed to find the source of heterogeneity for this analysis, and it was shown that mean age had a significant association with the result observed (P = 0.041). In addition, mean age contributed to the 44.05% of the heterogeneity observed. Sample size and female percentage did not have any association with the Gal-3 levels in COVID-19 cases (**Table 2**). The bubble plots for meta-regression based on sample size, mean age, and female percentage are shown in **Supplementary Figures 2–4**.

**Table 2:** Meta-regression of COVID-19 vs. controls and Severe vs. non-severe COVID-19 meta-analyses.

Moderator	No. of subjects		Meta-regression				R^2^ Analog (proportion of variance explained)
	Case	Control	Slope	95% CI		*p-value*	
COVID-19 vs. Control							
Sample Size	728	395	-0.002	-0.008	0.004	0.516	0%
Age (mean, years)	644	339	-0.055	-0.107	-0.002	0.041	44.05%
Female Percentage	572	385	-0.007	-0.098	0.084	0.883	0%
Severe vs. Non-Severe COVID-19							
Sample Size	231	339	0.0108	0.000	0.021	0.041	48.90%
Age (mean, years)	202	284	-0.028	-0.154	0.097	0.656	0%
Female Percentage	177	237	-0.040	-0.115	0.036	0.303	3.53%

CI, confidence interval; COVID-19, coronavirus disease of 2019.

The funnel plot for the assessment of publication bias is shown in **Supplementary Figure 5**, which showed apparent asymmetry and introduced the possibility of publication bias. However, Begg’s and Egger’s statistical tests did not show any significant publication bias (P = 0.367 and P = 0.186, respectively).

#### 3.3.2 Meta-analysis of Gal-3 levels in Severe vs. non-severe COVID-19

**Figure 4:**
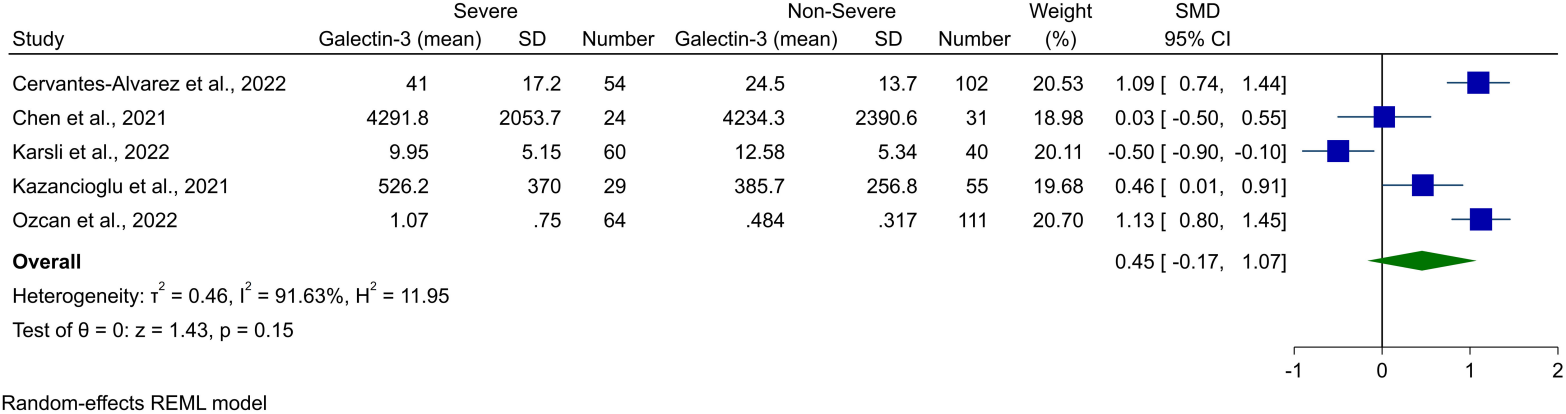
Forest plot for the meta-analysis of Gal-3 levels in severe vs. non-severe COVID-19.

Five studies reported Gal-3 blood concentrations in severe COVID-19 cases and compared it with non-severe patients. Meta-analysis for these levels showed a tendency toward higher levels of Gal-3 in severe instances of COVID-19; however, this was not significant (SMD 0.45, 95% CI -0.17 to 1.07, P = 0.15, **Figure 4**). High heterogeneity was observed for this analysis as well (*I^2^* = 91.63%). The Galbraith plot did not reveal any outlier study for this analysis (**Supplementary Figure 6**). However, in sensitivity analysis, removing Karsli et al. made the overall result significant (33).

In performing meta-regression for this meta-analysis, the sample size was found to have a direct association with the pooled effect size (P = 0.041). In addition, sample size and female percentage accounted for 48.90% and 3.53% of the observed heterogeneity, respectively (**Table 2**, bubble plots in **Supplementary Figures 7–9**).

Publication bias investigation based on the funnel plot inspection did not show any sign of bias (**Supplementary Figure 10**). In line, Begg’s and Egger’s statistical tests were not significant for publication bias (P = 0.227 and P = 0.178, respectively).

#### 3.3.3 Gal-3 levels’ association with COVID-19 complications

Intensive care unit (ICU) admission, need for ventilation, and death from COVID-19 had been assessed in the literature in association with Gal-3 levels. In a study by Kusnierz-Cabala et al. (34), the Gal-3 level was significantly higher in patients needing ICU treatment than those without it (4.77 [2.92-8.17] ng/ml vs. 2.30 [1.88-3.21] ng/ml, P = 0.001). Bruni et al. (29) study defined a composite endpoint of admission to ICU or 30-day mortality and found that COVID-19 cases with a composite endpoint had significantly higher levels of Gal-3 (12.1 [10.3-15.9] ng/ml vs. 8.1[6.5-11.3] ng/ml, P < 0.001). The outcome of death was the case investigated in studies by Portacci et al. (7) and Tawiah et al. (38). Portacci et al. reported median Gal-3 as 43.8 [36.2-59] ng/ml in dead patients, which was significantly higher than the ones with 30-day survival (21.9 [17.6-27.5] ng/ml, P < 0.001). In line, Tawiah et al. also measured higher Gal-3 levels in dead patients within 30 days of emergency department presentation. In a study conducted by Cannavo et al. (30), despite the observed higher mean value of Gal-3 serum levels in COVID-19 patients presenting cardiac function deterioration compared to those without cardiac complications, this difference was not statistically significant (Gal-3 levels between patients showing or non-showing cardiac complications: 4.7 ± 2.7 ng/mL vs. 3.5 ± 1.6 ng/mL, P = 0.35).

#### 3.3.4 Diagnostic and prognostic use of Gal-3

With an area under the curve (AUC) of 0.622, Karsli et al. (33) reported Gal-3 as a potential biomarker for the diagnosis of COVID-19. Prediction of ICU admission based on Gal-3 showed an AUC of 0.7 in the Portacci et al. study (7), and 0.802 in Ozcan et al. study (36). Moreover, the threshold of 15.51 ng/ml resulted in a sensitivity of 78% and specificity of 90% in the prediction of ICU admission, according to Kusnierz-Cabala et al. (34). The prediction of in-hospital mortality reported an AUC of 0.843 with sensitivity and specificity of 75% and 76.8%, respectively (36). The 30-day mortality prediction showed an AUC of 0.906 and 0.681 in studies by Portacci et al. (7) and Tawiah et al. (38). A study conducted by Rodríguez-Tomàs et al. (37) reported an AUC of 0.709 in differentiating COVID-19 patients from controls.

### 3.4 Gal-8 and COVID-19

Only one study has investigated Gal-8 levels in different COVID-19 severities (19). All severities of COVID-19 had significantly lower levels of Gal-8 in comparison with healthy controls, representing 8.6 (6.87-9.84), 5.49 (4.7-6.53), and 6.38 (5.65-7.04) for mild, severe, and critical COVID-19, respectively, in addition to 6.6 (6.12-7.86) for healthy controls.

### 3.5 Gal-9 and COVID-19

Five studies investigated the association between Gal-9 and COVID-19. Bai et al. (26) reported significantly higher levels of full-length Gal-9 in COVID-19 associated with pneumonia than in healthy control (P < 0.0001). In line with these results, two other studies reported higher levels of Gal-9 in SARS-CoV-2-infected individuals compared to non-infected ones (20, 28).

Further to these studies, Chen et al. (21) reported that patients with both severe and non-severe forms of COVID-19 had significantly higher levels of Gal-9 than controls (2044 [1385-3303] pg/ml, and 2811 [1866-4371] pg/ml vs. 739 [495-960] pg/ml, respectively).

For their part, Bozorgmehr et al. (28) confirmed the results above, showing an increase in Gal-9 in COVID-19 patients compared to healthy controls. In addition, these authors determined a cutoff of Gal-9 plasmatic concentration of 2,042 pg/ml that allowed the COVID-19 diagnosis with a sensitivity of 94.96% and specificity of 94.92%.

Finally, against this trend, the study from Yaşar et al. failed to find a statistical difference between mild COVID-19 and healthy controls (19). However, these authors found that severe and critical cases of the disease had significantly higher Gal-9 levels than patients with mild symptoms or healthy controls.

## 4 Discussion

Overall, our study demonstrated that the assessment of variation in circulating Gal-1, -3, -8, and -9 levels in response to SARS-CoV-2 infection could be used as potential diagnostic and prognostic non-invasive biomarkers of COVID-19.

In detail, our systematic review revealed that Gal-1 release is increased in COVID-19 patients compared to controls. However, the data about its relevance to disease severity was contradictory. For instance, in one study, it has been reported no significant difference between severe and non-severe COVID-19 patients (22). In comparison, another study reported a higher level of Gal-1 in critical cases compared to those with severe or mild COVID-19 (35).

Concerning Gal-3, our meta-analysis showed that the circulating levels of Gal-3 were significantly higher in COVID-19 patients than in healthy controls, with no significance between severe and non-severe cases. Moreover, our review support that Gal-3 levels are positively associated with COVID-19 complications. A huge body of literature indicates cytokine release as the main contributing factor to morbidity and mortality of COVID-19 (39). Gal-3 is also correlated with several inflammatory biomarkers, which may be the pathophysiology behind this capability to be a biomarker. Classic inflammatory biomarkers such as C-Reactive Protein (CRP), Ferritin, neutrophil count, and D-dimer have been shown to have associations with disease severity (40, 41); however, as stated by Cervantes-Alvarez et al., there is collinearity between them. In this study, after multivariate analysis, only CRP and Gal-3 were recognized as independent predictors of disease severity (31). Moreover, due to various cut-offs available for CRP, Gal-3 may even be a better candidate for measurement to stratify disease severity. However, it is worth noting that three reports are currently contrasting with this trend. For instance, Bruni et al. (29) stated that COVID-19 patients had insignificant levels of Gal-3 compared to controls. In line with this, Kusnierz-Cabala et al. (34) reported no significant difference in serum Gal-3 levels in COVID-19 patients compared with healthy controls. Meanwhile, Karsli et al. (33) reported that Gal-3 levels are lower in severe COVID-19 patients than in healthy controls.

In addition, despite limited, our systematic review of Gal-8 and -9 showed that lower Gal-8 and higher Gal-9 levels could be observed in COVID-19 patients compared with healthy controls. Concerning the correlation between Gal-8 and -9 to the disease severity, the data available are not conclusive. Indeed, our study showed a tendency toward higher levels in severe cases of Gal-9. Conversely, for Gal-8 the data are very limited as only one study investigated whether the serum level of this factor is differently modulated by COVID-19 severities (19). In this context, Yasar and colleagues reported a reverse trend of Gal-8 levels compared with other galectins. Nevertheless, it is worth noting that recent studies revealed Gal-8 binds exclusively to a specific motif of the viral receptor-binding domain (RBD) of SARS-CoV-2, called 30SLacNAc, while other galectins identify and bind to other additional motifs of the RBD as well (42). However, more studies are required to confirm this different trend seen in Gal-8 and look for possible explanations as well.

Together, these data suggest that further investigation into the clinical relevance and biological role of Gal-8 and -9 is needed. Indeed, understanding the role of these galectins will not only increase their diagnostic and prognostic significance but may also have therapeutic use in the fight against COVID-19 as well as other inflammatory disorders, as per Gal-1 and -3 which are currently considered two of the most critical therapeutic and drug target in various diseases (43). Studies regarding their use in COVID-19 patients showed that inhibition of Gal-3 could modulate the host immune response and reduce cytokine storm syndrome, a major cause of death in COVID-19, alongside preventing viral entry and reducing post-COVID-19 pulmonary fibrosis (44, 45). Also, different clinical trials are currently targeting Gal-3 for treating fibrosis in various diseases, such as idiopathic pulmonary fibrosis and non-alcoholic steatohepatitis-related liver fibrosis (13). In this context, a phase Ib/IIa randomized controlled platform trial is evaluating the potential effects of the Gal-3 inhibitor GB0139 in hospitalized patients with confirmed COVID-19 pneumonitis (46).

## 5 Limitations

There are some study limitations to note. First, Gal-1, -8, and -9 lacked sufficient original studies to perform a meta-analysis. Secondly, regarding the measurement methods, the studies used various techniques and equipment that consequently resulted in heterogeneity, which despite performing meta-regression, may add limitations. Finally, with the present data, we could not compare the effectiveness of galectins as diagnostic and prognostic tools to each other. Thus, additional studies are needed to compare different galectins not only with each other but also with other known COVID-19 biomarkers.

## 6 Conclusion

With the emergence of the COVID-19 pandemic, the healthcare systems now face a monumental challenge worldwide to find effective treatments and develop tools for disease diagnosis and monitoring disease progression. To our knowledge, this is the first comprehensive systematic review and meta-analysis evaluating the role of Gal-1, -3, -8, and -9 as potential serum and/or plasma biomarkers for the diagnosis and prognosis of COVID-19. Although we have identified several galectins differentially modulated in response to SARS-CoV-2 infection, our analysis supports a potential prognostic and diagnostic role for Gal-3, in particular, due to its elevated levels in patients compared with healthy controls and its positive correlation with the disease severity. However, additional studies are needed to corroborate our findings and further examine Gal-3’s levels in conjunction with other diagnostics or clinical parameters assessing host immunity, infection history, and other complications associated with SARS-CoV-2 infections. Similarly, further investigation is necessary to shed light on the role of the Gal-1, -8, and -9 that, based on the evidence provided here, may have a similar valuable diagnostic and prognostic role and be at the same time considered a potential therapeutic target, but that remain still poorly studied.

## Author contributions

AHB: Writing - original draft/Conceptualization/Formal analysis/Visualization. AKh: Writing - original draft, review and editing/Conceptualization/Supervision. SYA, AKa: Writing - original draft/Data curation. AC, CJD: Writing - review and editing. All authors read and approved the final manuscript.
