# Supplementary material for: Galectins can serve as biomarkers in COVID-19: A comprehensive systematic review and meta-analysis
Source: Front Immunol. 2023 Feb 23;14:1127247. doi: 10.3389/fimmu.2023.1127247 (PMC10009778; doi:10.3389/fimmu.2023.1127247)
Supplement: Supplementary file 1 [file DataSheet_1.docx]

**Supplementary Materials**

***Supplementary Table 1.*** *Search details*

| **Query** | | **Results**  **(26 Oct 2022)** |
| --- | --- | --- |
| **PubMed** | | |
| #1 | ("COVID-19"[tiab] OR "SARS-CoV-2"[tiab] OR "coronavirus"[tiab] OR "2019 nCoV"[tiab] OR "COVID-19"[Mesh]) | 313,524 |
| #2 | ("gal" OR "galectin*" OR "LGALS" OR "S-type lectin" OR "Galactose binding lectin" OR "galectins"[MeSH]) | 36,195 |
| #4 | #1 AND #2 | 197 |
| **SCOPUS** | | |
| #1 | (TITLE-ABS-KEY("COVID-19") OR TITLE-ABS-KEY("SARS-CoV-2") OR TITLE-ABS-KEY("coronavirus") OR TITLE-ABS-KEY("2019 nCoV")) | 440,073 |
| #2 | (TITLE-ABS-KEY("gal") OR TITLE-ABS-KEY("galectin*") OR TITLE-ABS-KEY("LGALS") OR TITLE-ABS-KEY("S-type lectin") OR TITLE-ABS-KEY("Galactose binding lectin")) | 44,313 |
| #4 | #1 AND #2 | 168 |
| **Web of Science** | | |
| #1 | (TS=("COVID-19") OR TS=("SARS-CoV-2") OR TS=("coronavirus") OR TS=("2019 nCoV")) | 375,552 |
| #2 | (TS=("gal") OR TS=("galectin*") OR TS=("LGALS") OR TS=("S-type lectin") OR TS=("Galactose binding lectin")) | 39,530 |
| #3 | #1 AND #2 | 104 |
| **Embase** | | |
| #1 | (‘coronavirus disease 2019’/exp OR ‘COVID-19’/exp OR ‘COVID-19’ OR ‘sars cov 2’/exp OR ‘SARS-CoV-2’ OR ‘coronavirus’/exp OR ‘2019 nCov’/exp OR ‘2019 nCov’ OR ‘coronavirus’) | 370,654 |
| #2 | ("gal" OR "galectin*" OR "LGALS" OR "S-type lectin" OR "Galactose binding lectin") | 52,444 |
| #3 | #1 AND #2 | 372 |

***Total: 841***

***After duplicate remove: 524***

***Supplementary Table 2****. Quality Assessment of Included Studies Based on Newcastle-Ottawa Scale (NOS)*

| **Study** | **Selection** | | | | **Comparability** | **Outcome** | | **Overall Score** |
| --- | --- | --- | --- | --- | --- | --- | --- | --- |
|  | **Representation** | **Sample size** | **Non-Respondents** | **Exposure** |  | **Outcome** | **Statistical test** |  |
| **Bai et al.** | * | * | * | ** | - | ** | * | 8 |
| **Bozorgmehr et al.** | * | * | * | ** | - | ** | * | 8 |
| **Bruni et al.** | * | * | * | ** | - | ** | * | 8 |
| **Cannavo et al.** | * | * | * | ** | - | ** | * | 8 |
| **Cervantes-Alvarez et al.** | * | * | * | ** | * | ** | * | 9 |
| **Chen et al.** | * | * | * | ** | - | ** | * | 8 |
| **De Biasi et al.** | * | * | * | ** | ** | ** | * | 10 |
| **Ercin et al.** | * | * | * | ** | - | ** | * | 8 |
| **Karsli et al.** | * | * | * | ** | ** | ** | * | 10 |
| **Kartal Baykan et al.** | * | * | * | ** | - | ** | * | 8 |
| **Kazancioglu et al.** | * | * | * | ** | - | ** | * | 8 |
| **Kusnierz-Cabala et al.** | * | * | * | ** | * | ** | * | 9 |
| **Markovic et al.** | * | * | * | ** | - | ** | * | 8 |
| **Ozcan et al.** | * | * | * | ** | - | ** | * | 8 |
| **Portacci et al.** | * | * | * | ** | - | ** | * | 8 |
| **Rodríguez-Tomàs et al.** | * | * | * | ** | - | ** | * | 8 |
| **Tawiah et al.** | * | * | * | ** | - | ** | * | 8 |
| **Yasar et al.** | * | * | * | ** | - | ** | * | 8 |

***
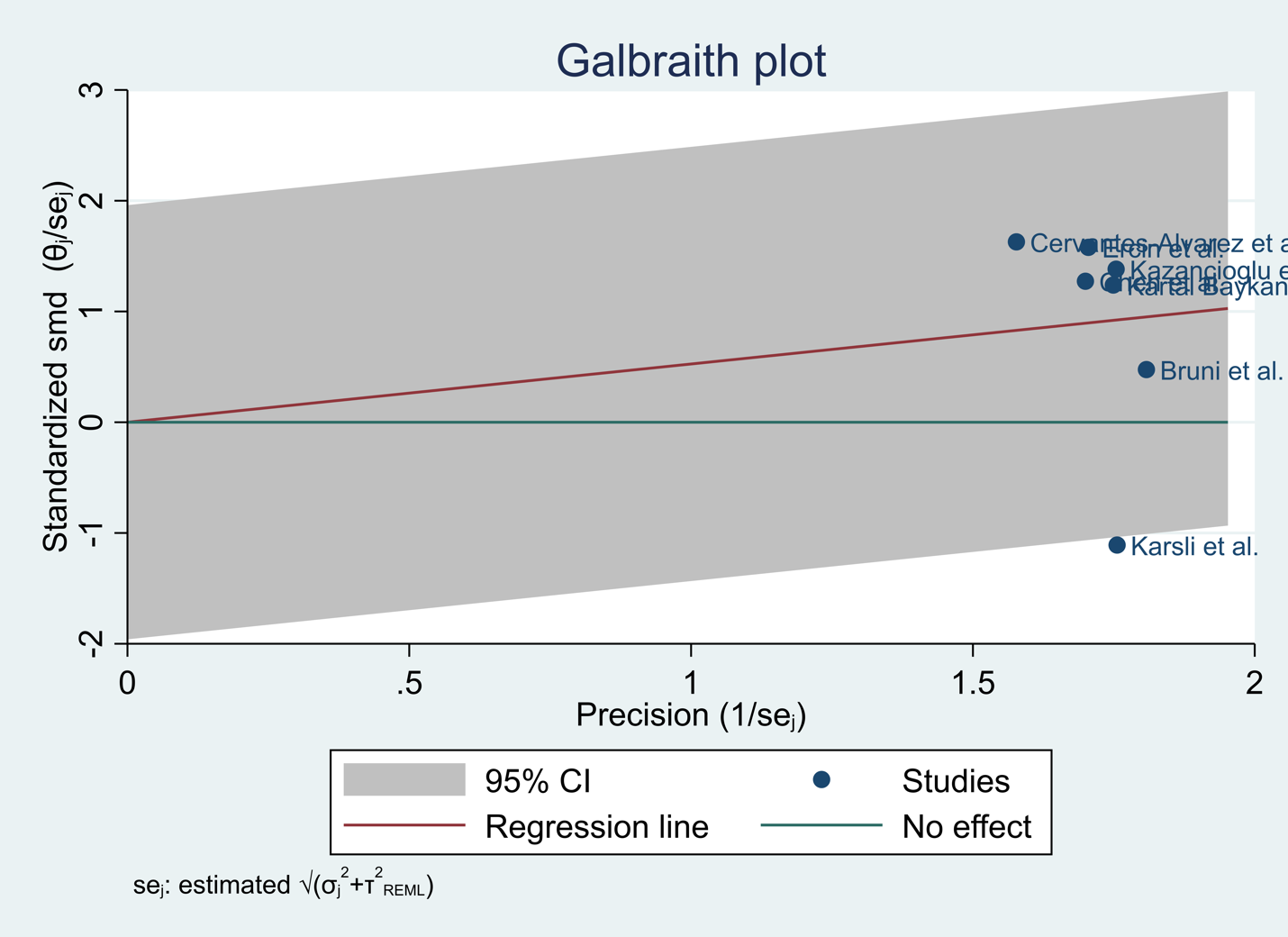
***

***Supplementary Figure 1.*** *Galbraith plot for meta-analysis of Galectin-3 levels in COVID-19 patients vs. healthy controls*

*
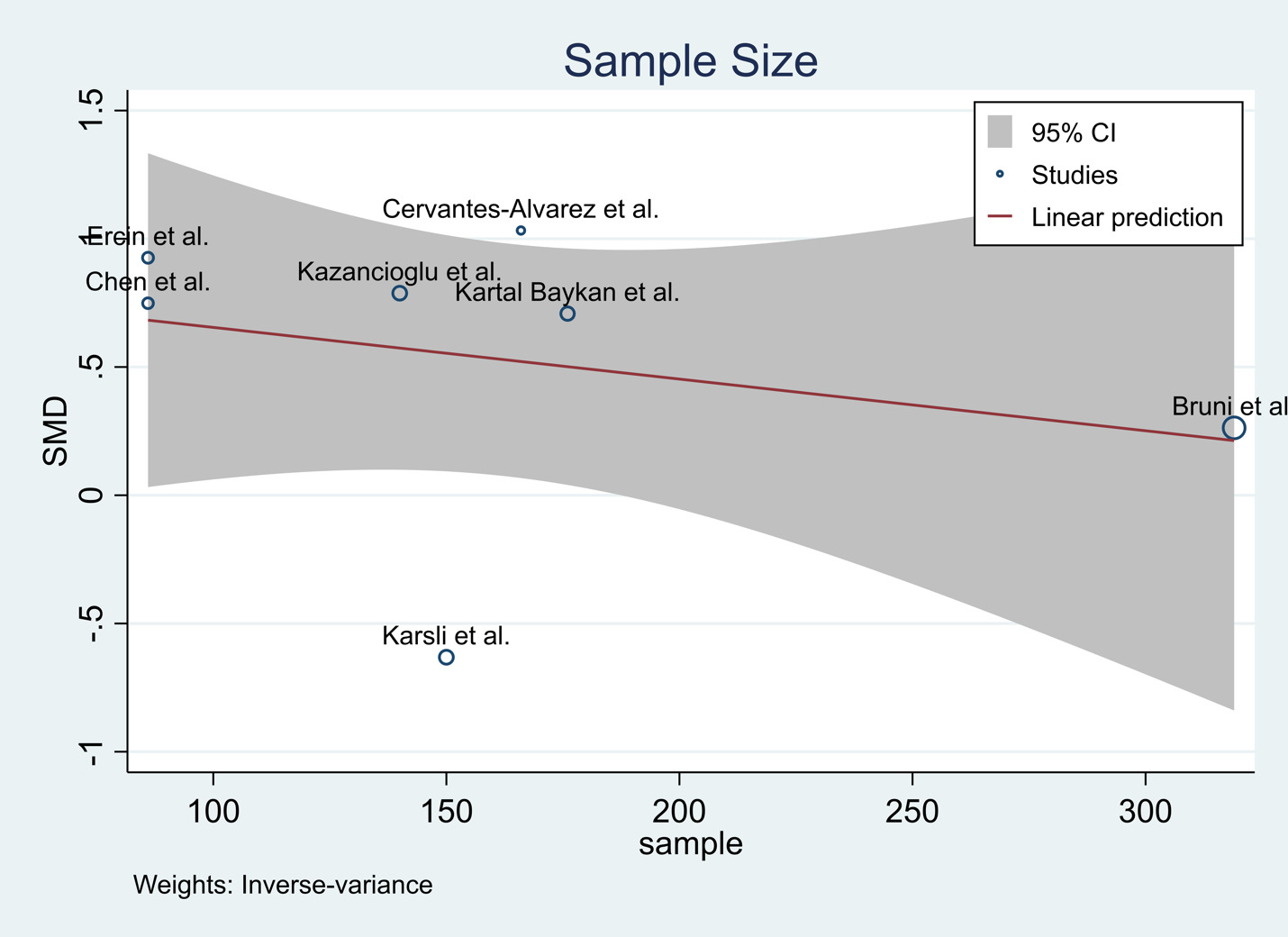
*

***Supplementary Figure 2.*** *Bubble plot of meta-regression based on sample size for meta-analysis of Galectin-3 levels in COVID-19 patients vs. healthy controls*

*
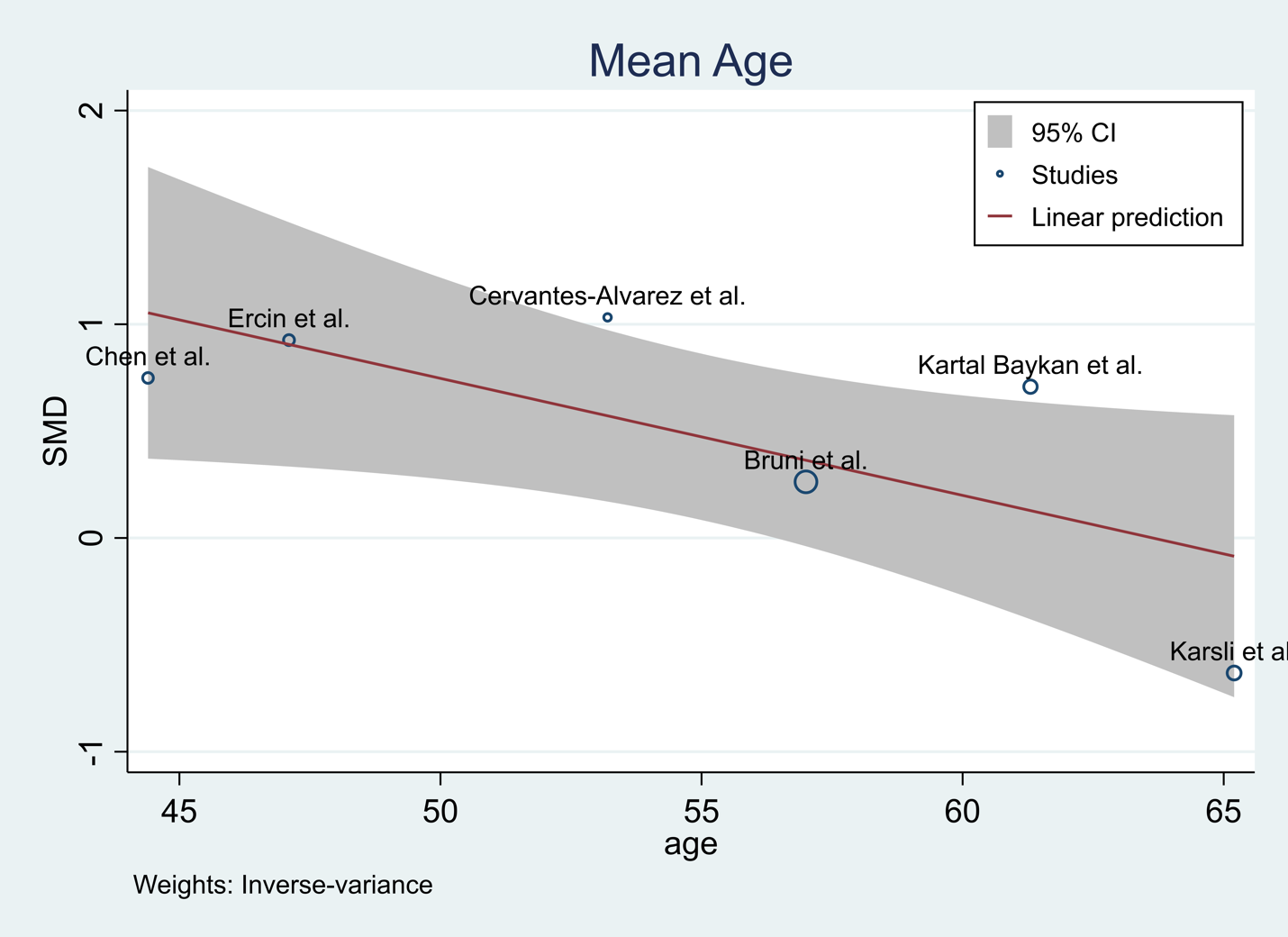
*

***Supplementary Figure 3.*** *Bubble plot of meta-regression based on mean age for meta-analysis of Galectin-3 levels in COVID-19 patients vs. healthy controls*

*
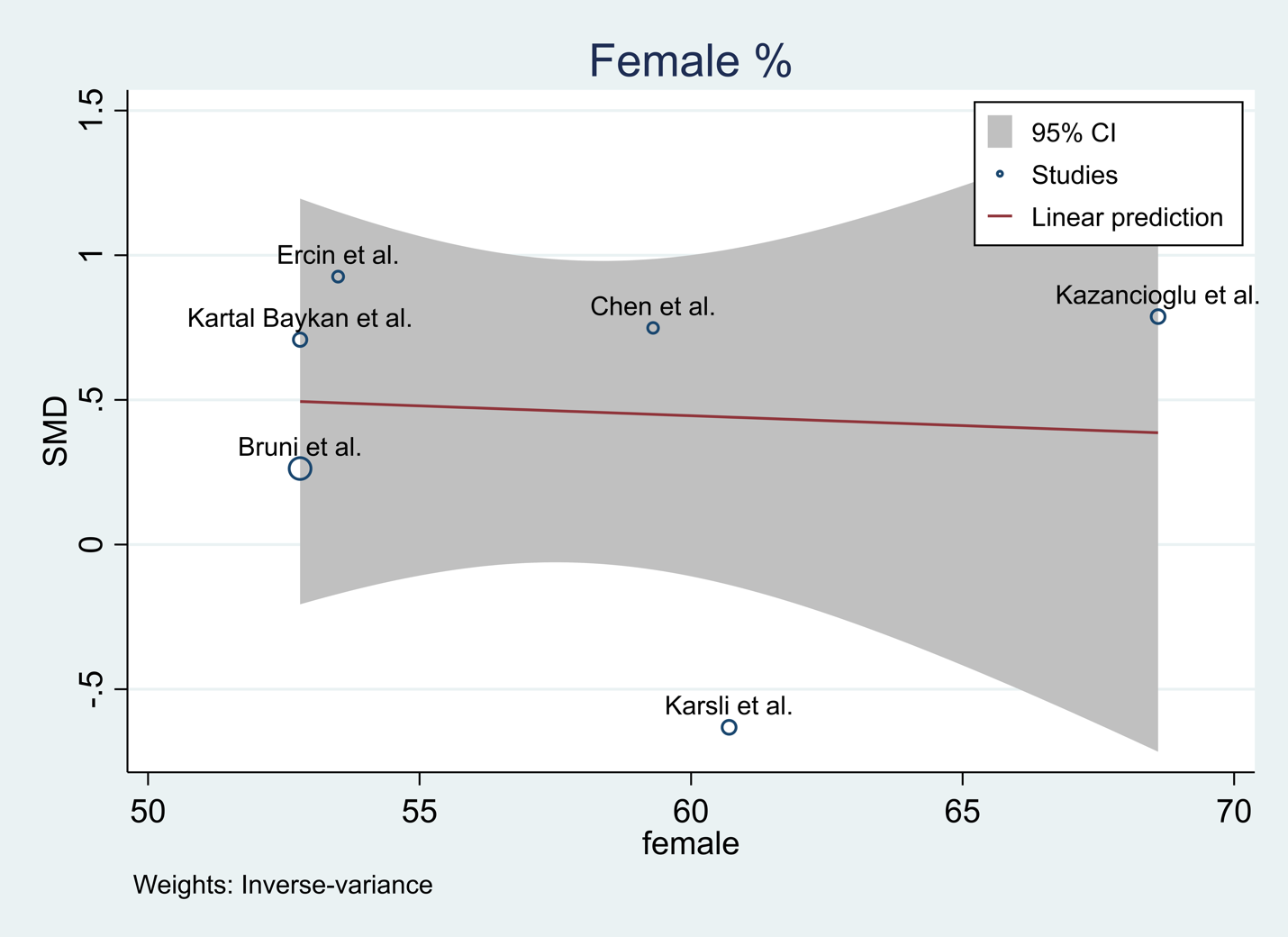
*

***Supplementary Figure 4.*** *Bubble plot of meta-regression based on female percentage for meta-analysis of Galectin-3 levels in COVID-19 patients vs. healthy controls*

*
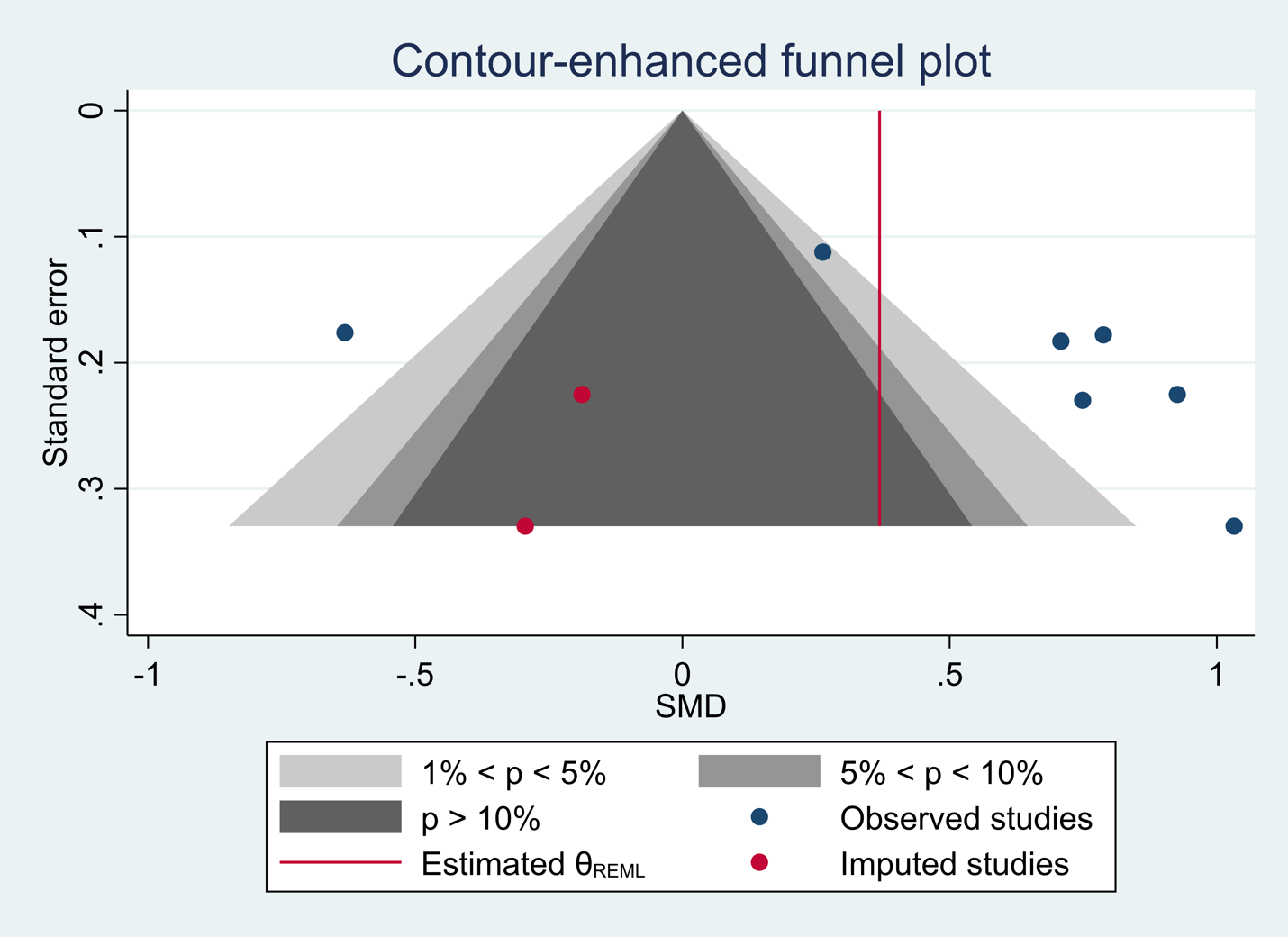
*

***Supplementary Figure 5.*** *Funnel plot for meta-analysis of Galectin-3 levels in COVID-19 patients vs. healthy controls*

*
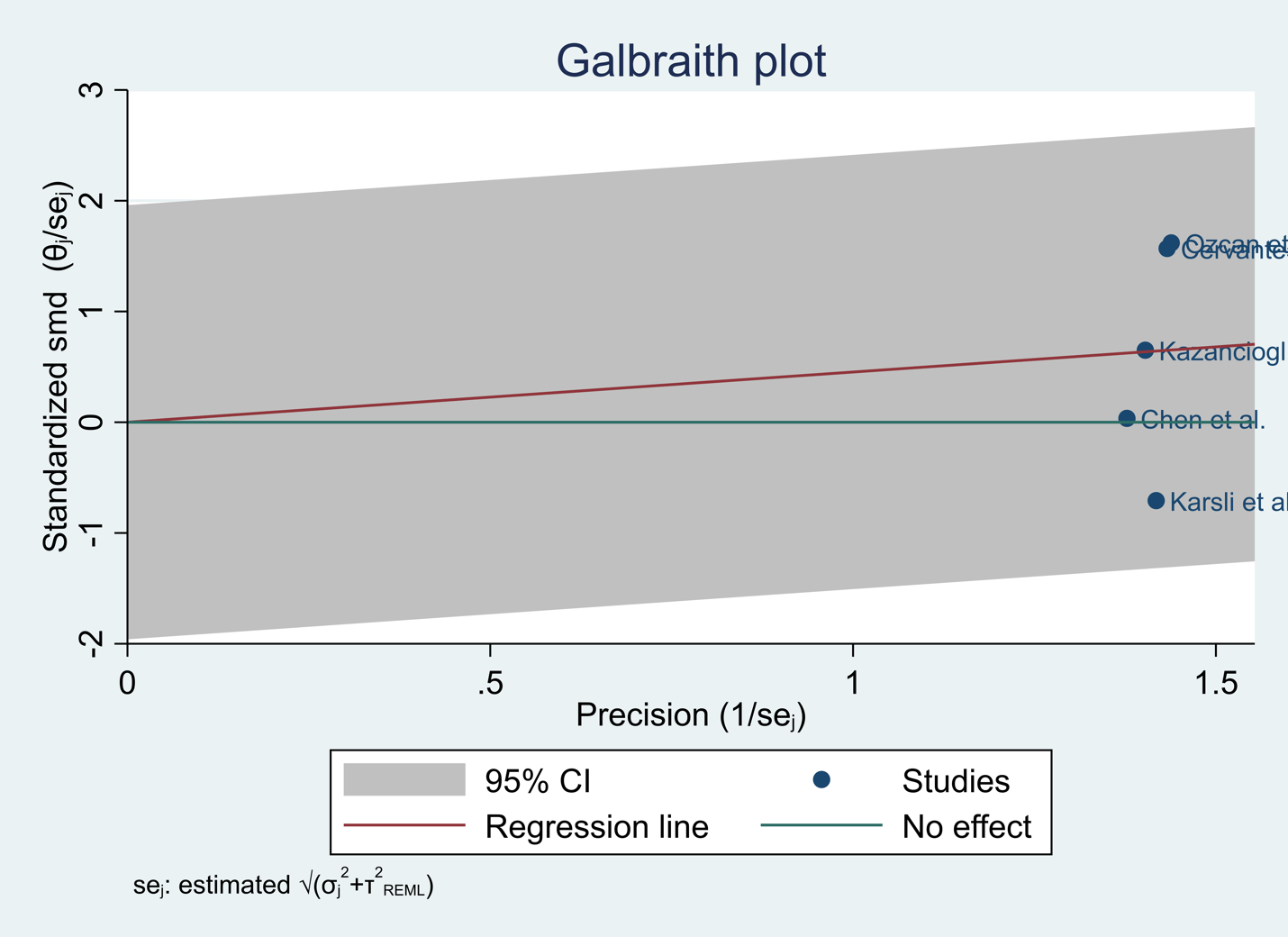
*

***Supplementary Figure 6.*** *Galbraith plot for meta-analysis of Galectin-3 levels in severe COVID-19 patients vs. non-severe patients*

*
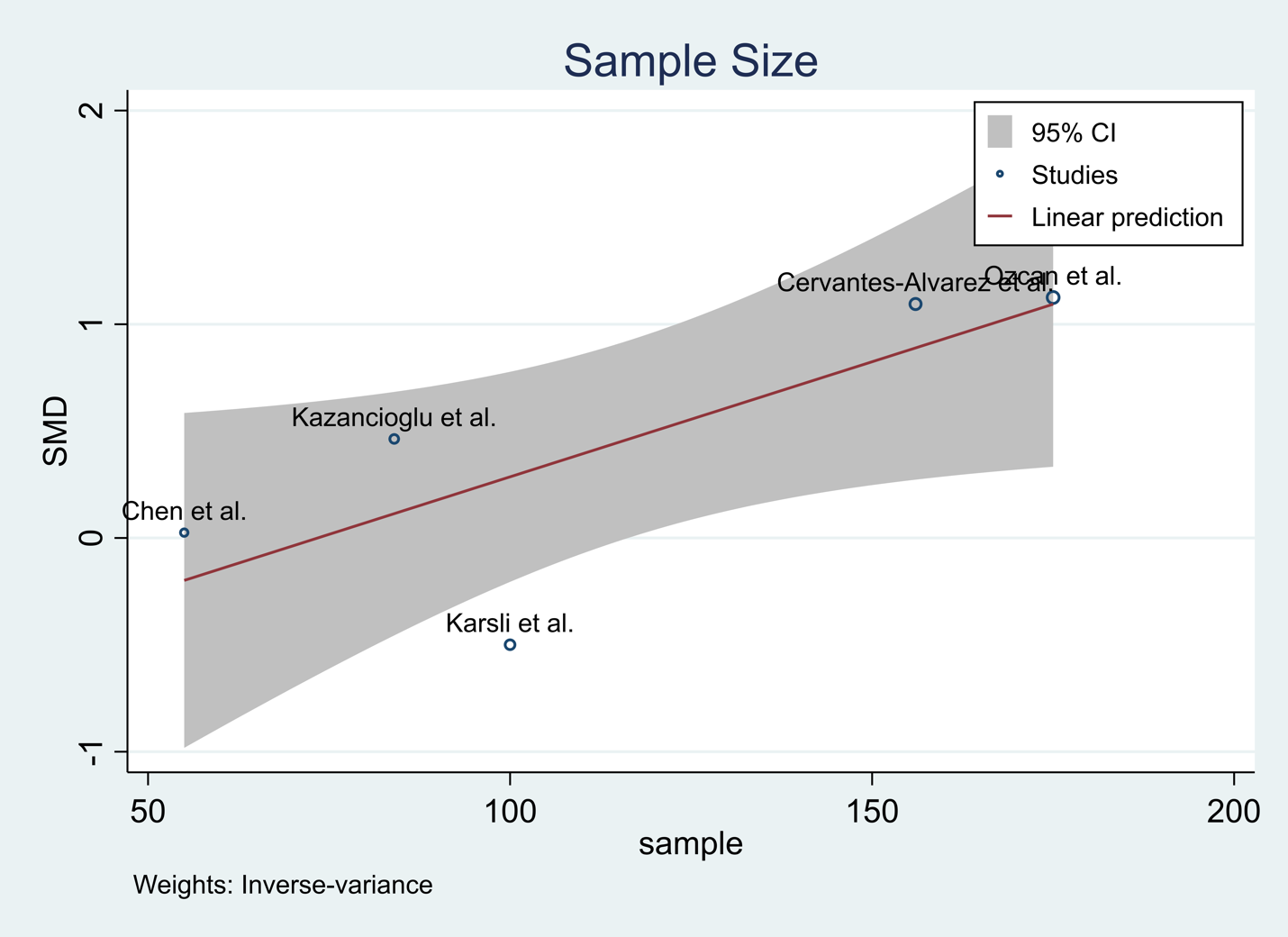
*

***Supplementary Figure 7.*** *Bubble plot of meta-regression based on sample size for meta-analysis of Galectin-3 levels in severe COVID-19 patients vs. non-severe patients*

*
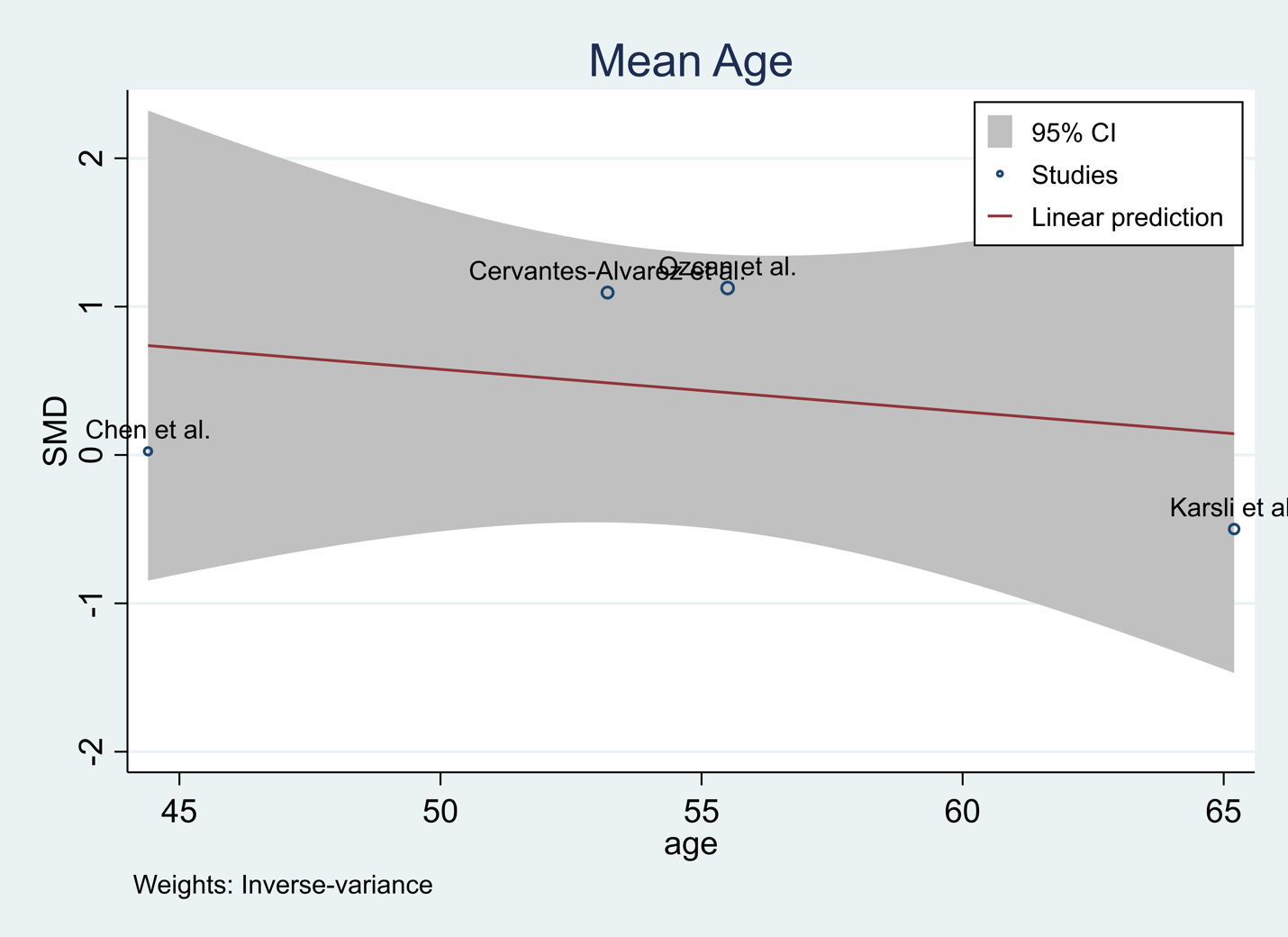
*

***Supplementary Figure 8.*** *Bubble plot of meta-regression based on mean age for meta-analysis of Galectin-3 levels in severe COVID-19 patients vs. non-severe patients*

*
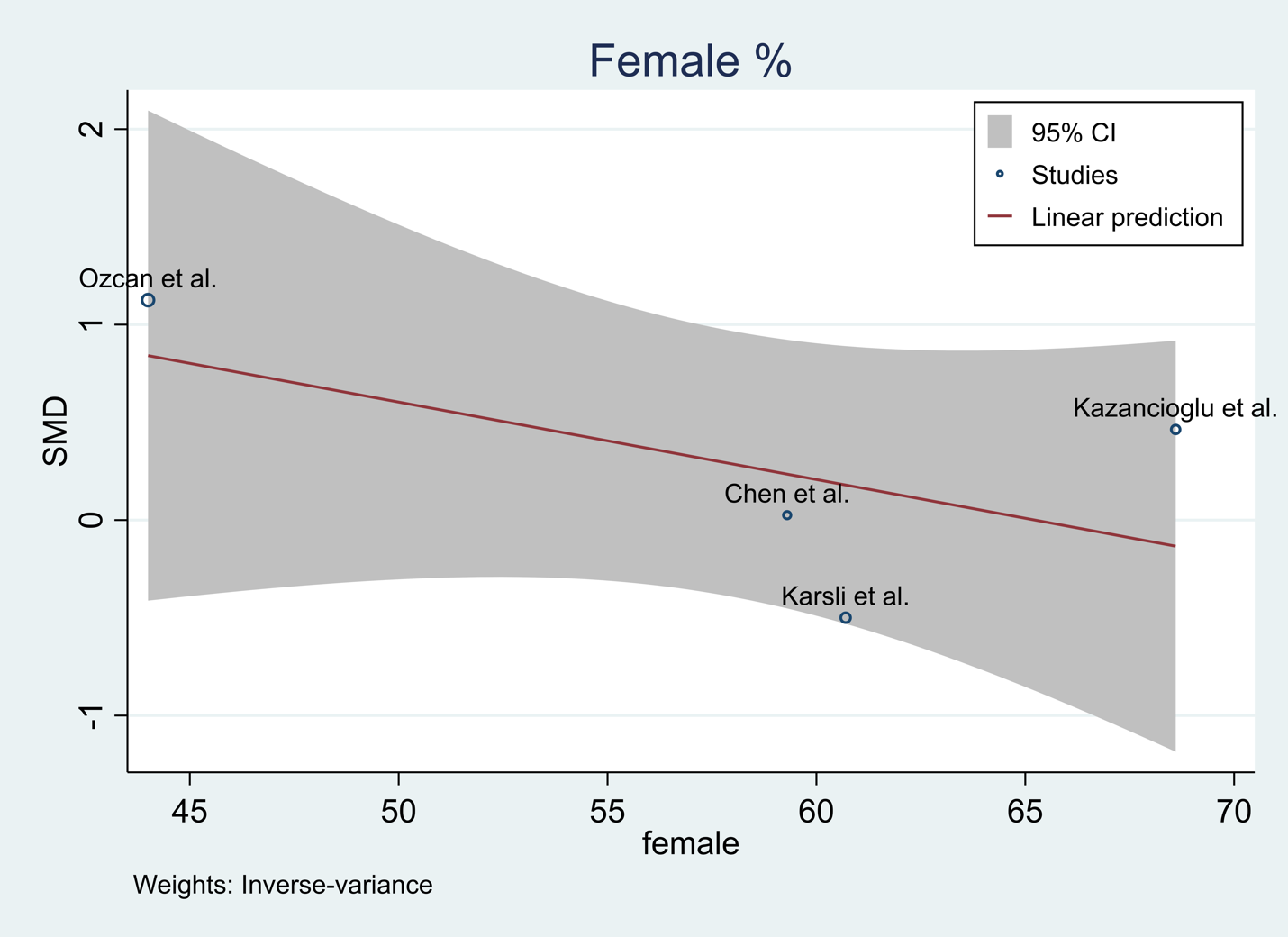
*

***Supplementary Figure 9.*** *Bubble plot of meta-regression based on female percentage for meta-analysis of Galectin-3 levels in severe COVID-19 patients vs. non-severe patients*

*
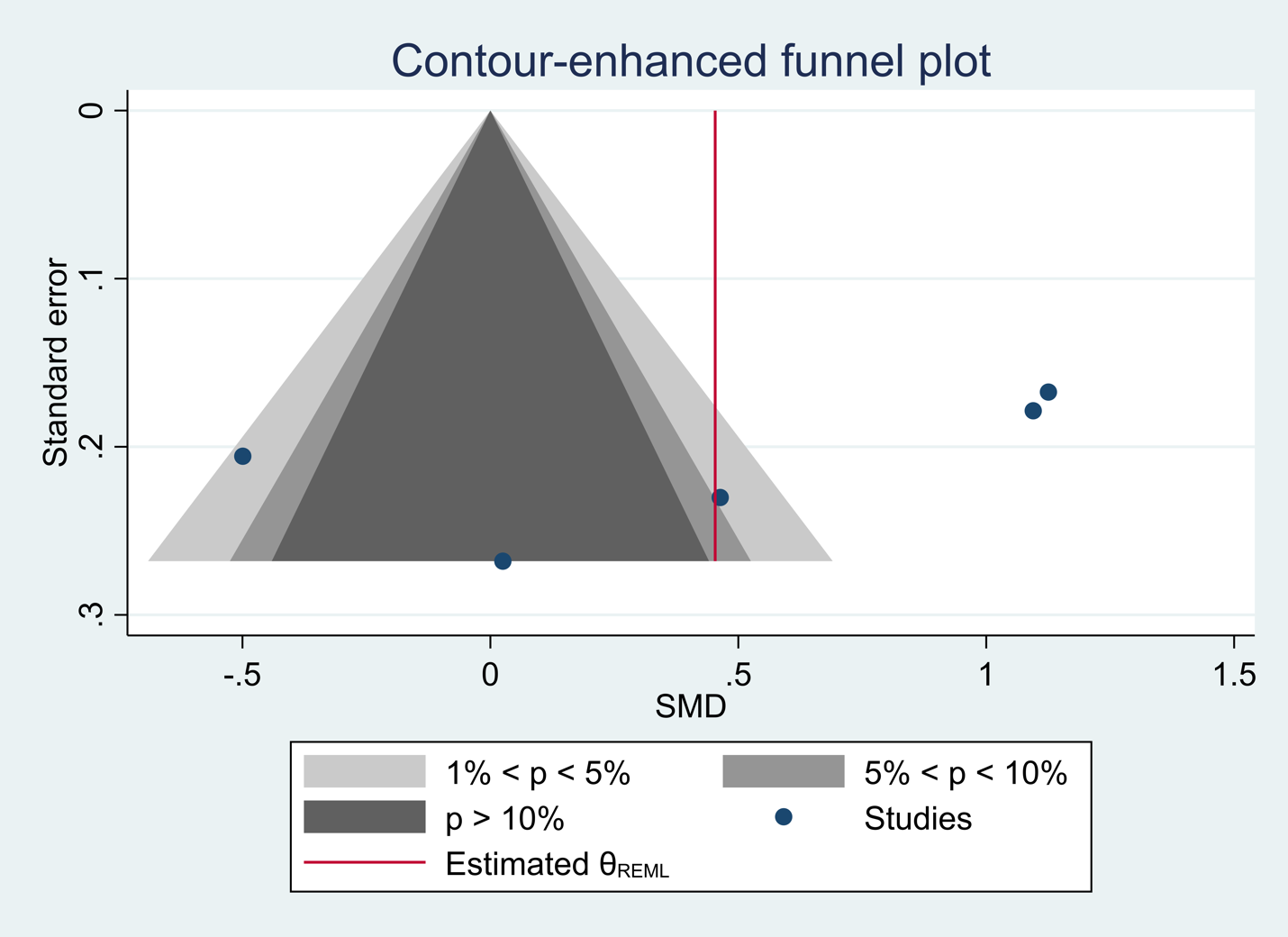
*

***Supplementary Figure 10.*** *Funnel plot for meta-analysis of Galectin-3 levels in severe COVID-19 patients vs. non-severe patients*
